# User Engagement and Weight Loss Facilitated by a Mobile App: Retrospective Review of Medical Records

**DOI:** 10.2196/42266

**Published:** 2023-01-24

**Authors:** Sarunas Valinskas, Marius Nakrys, Kasparas Aleknavičius, Justinas Jonusas, Angelė Lileikienė

**Affiliations:** 1 KiloHealth Vilnius Lithuania; 2 Lithuania Business University of Applied Sciences Klaipėda Lithuania

**Keywords:** intermittent fasting, fasting, weight, weight loss, mobile application, body composition, mHealth, mobile health, diet, dietary intervention, weight loss outcome, adherence, engagement, mobile app, motivation, intervention outcome, fasting apps, dietary interventions, obesity, regression analysis

## Abstract

**Background:**

Intermittent fasting (IF) has gained popularity in recent years for its effect on weight loss and supposed additional health benefits, such as a positive effect on body composition and metabolic markers. Mobile apps can act as platforms that help deliver dietary interventions by improving adherence and motivation. Although the effect of IF on weight loss has been demonstrated in earlier trials, there is not much research about the engagement and weight loss results with IF apps.

**Objective:**

Our main objective was to compare how a nudging platform (including smart scales) influences engagement (the extent to which users interact with the app measured by the number of active days) with the app among users who had obesity at the beginning of use. The secondary objectives were to evaluate the body weight changes among active and nonactive users and, finally, to evaluate the body composition changes of users possessing smart scales during app usage. Through this study, we hope to provide (1) more insight into how nudging (using smart scales as a nudging platform) is associated with engagement with the mobile app, (2) how engagement with the mobile app is associated with weight loss, and (3) how IF is associated with body composition.

**Methods:**

We performed a retrospective analysis of data from 665 users with obesity (BMI≥30) who started using the IF app DoFasting. Of them, 244 used body composition scales that estimated body fat and body muscle values. Users were stratified into engagement groups in accordance with their activity ratio (number of active days divided by the total time of use). Baseline and final users' weight (in kg), body fat (in %), and body muscle (in %) were compared.

**Results:**

Our findings suggest an association between the nudging platform (smart scales) and better engagement with the app. Smart scale users had a significantly higher activity ratio than regular users. Additionally, active DoFasting users lost significantly more weight. Further, body composition analysis showed that app usage might be related to body fat loss and an increase in muscle mass.

**Conclusions:**

We found a possible association between the nudging and gamified elements and higher app engagement. Additionally, increased app engagement is associated with increased weight loss. Thus, nudging and gamified elements of mobile health apps, such as interactive tools, goals, challenges, and progress tracking, are suggested to affect engagement positively and should be investigated further in future research. Finally, the IF regime delivered through the DoFasting app might be related to the body muscle mass gain and reduced fat mass.

## Introduction

In recent years, intermittent fasting (IF) has become a fairly popular diet option for its supposed benefits to health and, most importantly, its effect on weight loss [1]. The most popular IF plans for weight loss are the 5:2 regime, alternate-day fasting, and time-restricted feeding [2-5]. Whatever the fasting regime, IF has been noted to help lose weight, mainly because it reduces overall caloric intake [6]. A review of IF intervention trials has found that most fasting regimes help lose weight [7]. However, the question remains whether IF is superior to regular caloric restriction (CR) regimes. A meta-analysis by Harris et al [8] found that for short-term weight loss in adults with overweight or obesity, IF and CR regimes yield similar results.

Nonetheless, there are data suggesting that IF may have additional benefits. Some human studies have shown that with a similar reduction of caloric intake as in a CR diet, IF can result in more weight loss, better insulin sensitivity, and improved cholesterol levels [9-11]. Perhaps most importantly, several studies report that weight loss with IF results in more fat loss while sparing muscle mass [12,13]. For example, Welton et al [12] have noted in a systematic review of IF studies that the majority of weight loss with IF is due to fat loss. Additionally, in a randomized pilot study, Catennaci et al [14] found that after a 24-week follow-up, weight regains in an intermittent fasting group were limited to lean muscle, compared to fat mass in a CR group. This would make IF particularly attractive for weight loss. However, more studies about body composition changes with IF are still needed.

Facilitating diets with mobile apps is increasingly popular. Features such as reminders, feedback, setting of goals, and availability of diet-related educational information allow mobile apps to be platforms that help maintain dietary interventions [15,16]. Studies have shown that adherence to diets and self-evaluated motivation are better when mobile apps are used to follow a diet plan [17,18]. Carter et al [17] showed that adherence to the 6-month–long intervention was significantly higher in the smartphone user group (93%) than in the website (55%) or the diary (53%) user groups. Additionally, a cross-sectional survey completed by West et al [18] showed that majority of participants (54.4%) linked usage of nutrition apps and changes to their eating behavior.

Better adherence and higher motivation may thus yield better weight loss results. In addition, it has been shown that actively engaging with a mobile app is associated with losing a significant amount of weight among all BMI groups (*P*<.001) [19]. Nonetheless, research on how to best engage users is still relatively scarce, and optimizing the benefits that mobile apps bring is an ongoing effort. Some researchers suggest that “gamified” features of health apps, such as challenges, appeal to users and should be explored further [20]. Additionally, it has been shown that nudging helps increase adherence and retention to the intervention [21]. Smart trackers (such as bracelets and smart scales) work as nudging platforms, incorporating nudging techniques such as automatization, user-friendly data presentation, and others [22,23].

In this study, we retrospectively analyzed the data from users of the mobile app DoFasting, which is aimed at helping follow an IF regime. Our main objective was to compare how the nudging platform (such as smart scales) influences the engagement to the app of the users who had obesity at the beginning of use. The secondary objectives were to evaluate the body weight changes among active and nonactive users and, finally, to evaluate the body composition changes of users possessing smart scales during app use. Through this, we hope to provide (1) more insight into how nudging (using smart scales as a nudging platform) is associated with engagement with the mobile app, (2) how engagement with the mobile app is associated with weight loss, and (3) how IF is associated with body composition.

## Methods

### Study Design and Recruitment

We have performed a retrospective review of medical records. The initial study cohort comprised 1141 consecutive users who started using the DoFasting app between November 1, 2020, and January 1. 2021. After the initial data screening, only users with obesity were retained (N=665). Additionally, in order for users to be included in the study, they had to have at least 2 weight measures during app usage. In total, 421 users measured their weight with regular scales, and 244 had smart scales. There were 268 males and 397 females in the final cohort.

### Measurement of Engagement

The number of active days (AD) was obtained from the app's login data. Active days were those when users completed meaningful actions in the app, such as logging a meal, completing a workout, reading educational content, logging their calorie or water intake, or completing a challenge. Total time of use (TT) was calculated by subtracting the first login date from the last login date. The ratio between AD and TT was used later to analyze engagement. Users were divided in the active and nonactive groups later on using this ratio. Active users were defined as those whose AD and TT ratio (activity ratio) was higher than or equal to 0.5, while nonactive users were those whose AD and TT ratio was lower than 0.5.

### Measures

To measure the users' body composition, we used DoFasting smart scales (FCC ID: 2AVEN-CF516), which have 4 biometrical impedance analysis sensors. For this study, we used body fat (in %) and body muscle (in %) data from the smart scales.

Gender, baseline body weight, and BMI values were self-reported by users during the account setup stage. The final weight value was self-reported in the group without smart scales (regular users), while the final weight and body composition values were automatically obtained from the smart scales in the group of users that used it (Table 1). BMI was calculated using the standard formula. Five BMI classes were determined in accordance with the Centers for Disease Control and Prevention’s standards: ≥18.5 and <25, healthy weight; ≥25 and <30, overweight; ≥30 and <35, obesity class I (OCI), ≥35 and <40, obesity class II (OCII), and ≥40, obesity class III (OCIII).

**Table 1:** Descriptive statistics of acquired data.

			Males		Females
**Smart scales, n**					
	With	106		138	
	Without	162		259	
Active days, mean (SD)			33.03 (20.28)		27.61 (20.10)
Total time, mean (SD)			55.71 (23.35)		53.00 (27.86)
Activity score, mean (SD)			0.42 (0.33)		0.39 (0.31)
**Weight at baseline (kg), mean (SD)**					
	Obesity class I	104.79 (7.68)		87.75 (8.47)	
	Obesity class II	119.78 (11.31)		100.79 (9.67)	
	Obesity class III	140.03 (12.66)		115.24 (10.79)	
**Body fat mass at baseline (%), mean (SD)**					
	Obesity class I	32.24 (3.95)		36.95 (4.92)	
	Obesity class II	36.46 (3.78)		38.53 (4.33)	
	Obesity class III	41.75 (3.51)		43.18 (3.81)	
**Body muscle mass at baseline (%), mean (SD) **					
	Obesity class I	60.43 (3.88)		55.75 (4.87)	
	Obesity class II	56.40 (3.72)		54.37 (4.29)	
	Obesity class III	51.25 (3.48)		49.89 (3.77)	

### Procedures

GraphPad Prism (version 9; GraphPad Software Inc), was used to analyze the obtained data. Nonparametric tests were used as it was found that all data were skewed and kurtotic, and the Shapiro-Wilk test confirmed that the data samples were distributed nonnormally. A 1-way ANOVA was used for multiple comparisons. The value of significance was chosen to be .05.

### Ethical Considerations

The investigation was performed in accordance with the ethical standards of the institutional review board (BRANY approved this retrospective chart review study in June 2022; registration ID.: 22‐08‐034‐939) and with the 1964 Helsinki Declaration and its later amendments. All participants agreed that their depersonalized data would be used for scientific purposes during the DoFasting account creation phase. As all data are depersonalized, there are no possibilities for the authors to retrace the identities of the participants. Participants received no compensation for their participation in the study.

## Results

The activity ratio was significantly higher among users using smart scales (Table 2) in all weight groups except for the male OCIII group. However, the statistical tendency was visible there (*P*=.09).

Later, users were stratified into active and nonactive groups in accordance with the methodology described earlier. There was a significant difference between the usage of smart scales and being more engaged with the app among male and female cohorts in all weight groups except in males with third-degree obesity (Table 3). Although the results were not significant, the tendency was visible in this group (*P*=.06).

Additionally, body weight was significantly lower in the active users group than in the nonactive users group among males and females, except for the OCII female group, where only a tendency was found (*P*=.07). Active users lost more weight than nonactive ones (Table 4).

When the users using smart scales were assessed, it was found that there was a change in body weight, body fat, and body muscle mass among male and female users in all weight groups (Table 5). Additionally, when active users were compared to nonactive users, it was found that active male users in the OCI and OCII groups lost more weight and body fat and gained more muscle mass than their counterparts in the nonactive group. The same was observed in the female OCI group.

**Table 2:** Comparison of activity score between users who have smart scales and regular users.

	Activity scores of males				Activity scores of females		
Weight group	Regular, mean (SD)	Smart scales, mean (SD)	*P* value	Regular, mean (SD)		Smart scales, mean (SD)	*P* value
Obesity class I	0.28 (0.27)	0.56 (0.30)	<.001	0.32 (0.27)		0.52 (0.30)	<.001
Obesity class II	0.30 (0.29)	0.69 (0.26)	<.001	0.29 (0.26)		0.54 (0.31)	<.001
Obesity class III	0.33 (0.35)	0.65 (0.29)	.009	0.32 (0.28)		0.65 (0.29)	<.001

**Table 3:** User distribution in the activity groups according to their usage of smart scales.

Weight group and activity group		Males			Females		
		Regular, n	Smart scales, n	*P* value	Regular, n	Smart scales, n	*P* value
**Obesity class I**				<.001			.002
	Active	15	36		32	32	
	Nonactive	81	29		91	34	
**Obesity class II**				<.001			<.001
	Active	11	22		15	26	
	Non-Active	37	6		71	20	
**Obesity class III**				.06			<.001
	Active	5	8		13	18	
	Non-Active	13	5		37	8	

**Table 4:** Comparison of weight loss between active and nonactive users.

	Weight loss among males (kg)			Weight loss among females (kg)		
Weight group	Active, mean (SD)	Nonactive, mean (SD)	*P* value	Active, mean (SD)	Nonactive, mean (SD)	*P* value
Obesity class I	–3.36 (2.82)	–2.10 (2.65)	.003	–2.40 (2.66)	–1.00 (2.92)	.002
Obesity class II	–5.26 (5.72)	–1.59 (3.02)	.001	–2.72 (3.34)	–1.27 (2.10)	.07
Obesity class III	–5.25 (4.94)	–3.71 (3.12)	.048	–3.74 (3.35)	–1.60 (2.23)	.002

**Table 5:** Observed differences in body weight, fat, and muscle mass between active and nonactive users.

Weight group		Males			Females		
		Active	Nonactive	*P* value	Active	Nonactive	*P* value
**Obesity class I (n=131)**							
	Participants, n	36	29	N/A^a^	32	34	N/A
	Body weight (kg), mean (SD)	–3.40 (3.11)^b^	–1.48 (2.60)^b^	.02	–2.03 (2.22)^b^	–1.37 (3.37)^b^	.02
	Body fat (%), mean (SD)	–0.91 (0.76)^b^	–0.48 (0.64)^b^	.009	–0.44 (0.73)^b^	–0.36 (0.97)^b^	.09
	Body muscle (%), mean (SD)	0.86 (0.73)^b^	0.43 (0.62)^b^	.005	0.40 (0.68)^b^	0.37 (0.90)^b^	.16
**Obesity class II (n=74)**							
	Participants, n	22	6	N/A	26	20	N/A
	Body weight (kg), mean (SD)	–5.31 (5.00)^b^	–2.70 (2.24)^c^	.02	–1.73 (2.10)^b^	–1.60 (3.68)^c^	.82
	Body fat (%), mean (SD)	–0.77 (0.98)^b^	–0.10 (0.11)^c^	.02	–0.37 (0.46)^b^	–0.19 (0.26)^c^	.30
	Body muscle (%), mean (SD)	0.72 (0.93)^b^	0.17 (0.21)^c^	.02	0.38 (0.48)^b^	0.16 (0.19)^c^	.22
**Obesity class III (n=39)**							
	Participants, n	8	5	N/A	18	8	N/A
	Body weight (kg), mean (SD)	–5.55 (5.24)^b^	–5.49 (3.98)^b^	.88	–2.42 (2.89)^b^	–1.60 (2.25)^b^	.27
	Body fat (%), mean (SD)	–0.51 (0.39)^b^	–0.58 (0.36)^c^	.66	–0.33 (0.55)^b^	–0.39 (0.29)^b^	.56
	Body muscle (%), mean (SD)	0.51 (0.38)^b^	0.54 (0.32)^c^	.83	0.32 (0.52)^b^	0.35 (0.29)^b^	.72

^a^N/A: not applicable.

^b^Significant at *P*<.05.

^c^Tended toward significance at *P*<.09.

## Discussion

### Principal Findings

Our main findings are that there may be an association between the nudging platform (smart scales) and better engagement with the app. The activity ratio was significantly higher among users using smart scales. Additionally, active DoFasting users lost significantly more weight than nonusers. Further, body composition analysis showed that app usage might be related to body fat loss and an increase in muscle mass.

Liu et al [16] noted certain features of mobile apps that may be useful for managing weight-related conditions: goal setting, reminders, feedback, educational materials, and visualizations. Other researchers argue that through these features, mobile apps can be facilitators of dietary behavior changes [18]. DoFasting tracks its users’ fasting cycles and provides reminders accordingly. Educational materials about IF are available in an articles section. Furthermore, the users are able to set personalized goals, take up challenges, and visualize and track their progress metrics. These latter features are particularly important because they represent gaming elements within the app. Although many IF apps are available, studies indicate that they often lack much needed properties of gamification that could entice users and keep them engaged in the longer term [24]. Including challenges and tracking of progress can bring a certain appeal and improve self-management with the app [20]. As a side note, we also noticed that having a smart scale predicted being more engaged within the app (Tables 2 and 3). In a way, purchasing and integrating body composition scales into weight loss further gamifies the process by providing more metrics to track and goals to set, thus increasing engagement and, consequently, weight loss.

Some earlier studies have found that engagement with the mobile app is an important predictor of weight loss [25,26]. Kim et al [25] showed that there was a significant positive correlation between BMI change and a normative influence on other app users, and a number of red articles, posts, and responses while using the weight loss app (correlation coefficients were as follows: 0.28, 0.28, 0.34, and 0.18; *P*<.01). Furthermore, Michaelides et al [26] reported corresponding results: an app, designed for individuals with diabetes, users who logged their meals, and those who posted in group chats were associated with significant weight loss (*F*_1,41_=8.84, *P*=.005 for group posts and *F*_1,42_=4.99, *P*=.03 for meal logging). This study reports similar results. Active users were associated with significantly higher weight loss than nonactive users among all weight groups (Table 4). It may be that actively engaging with the features of the DoFasting app helped its users lose more weight. Alternatively, those who were more motivated and successful in losing weight may have also appeared more active in the app. Most likely, the true effect lies in between—motivated users engaged with the app, which, in turn, helped them retain their motivation, track their progress, and achieve better results.

A possible benefit of IF might be its favorable effect on body composition. A systematic review by Vitale and Kim [27] suggests that there is strong evidence that IF can improve body composition measures. Studies indicate that after 12 weeks of IF, an average loss of 2% of body fat can be expected [27]. Similarly, in a systematic review, Guerrero et al [28] found that some studies showed a higher fat mass reduction with IF compared to that with regular energy restriction diets. Our results agree with those of previous studies. In our cohort of users who had smart scales, we found that IF may be associated with body weight, fat loss, and muscle mass gain among males and females. Additionally, active males in OCI and OCII groups and females in the OCI group lost significantly more weight than their counterparts in the nonactive group (Table 5). Interestingly, such an effect was not visible among females in the OCII group. Our results agree with those of a systematic review, where it was found that males tend to lose weight more easily [29]. However, more research is still required about what metabolic changes IF brings about and how they affect body composition.

Many fasting studies, although considered “short-term,” analyze fasting intervals that are much longer than those used in IF, so their results may not always be applicable [30]. It has been noted that in starvation of lengths similar to those in IF, fatty acid use as an energy source increases: in the first 24 hours of fasting, lipolysis and oxidation of fats increase considerably [31]. Additionally, short fasts may help preserve body proteins. Some fasting studies show that there are no indications of muscle proteolysis after the first 36 hours of fasting, and alternate-day fasting does not increase protein metabolism in healthy adults [32,33]. Including the data from users with body composition scales allowed us to analyze changes in their body composition under IF. Consumer devices for bioelectrical impedance analysis are generally considered accurate for field studies [34]. Although their estimates are not as accurate as those of magnetic resonance imaging or dual X-ray absorptiometry, given the nature of this study, body composition scales were the next best option. It appeared that a significant amount of weight lost by DoFasting users was fat mass, not just water weight. Additionally, the percentage of body muscle was not reduced in any of the groups and increased by up to 0.84% in the male OCI group. These results agree with what was discussed above—IF may help lose weight by reducing fat mass and preserving body proteins. We thus suggest that IF weight loss studies gather and analyze body composition data.

One strength of this study is adding to the scarce body of research about engagement with mobile apps. Additionally, we have shown with a relatively large sample that IF, with the help of the DoFasting app, was effective for weight loss in the short term. Furthermore, assigning users to engagement groups allowed us to analyze how engagement predicted weight loss, stressing its importance for mobile app–based interventions. Finally, including body composition scales in the study helped track fat mass and body muscle changes under short-term fasting regimes.

### Limitations

The retrospective nature of the analysis is one of the limitations that does not let us currently derive firm conclusions about the effects of the DoFasting app. A prospective study with an established control group would help more strongly establish the effectiveness of IF with mobile apps. In addition, it is unclear in this study how sustainable the observed weight loss is. Some research suggests that although weight loss results with mobile app use are significant, they tend to lessen as time passes [35]. In the future, having data from longer time intervals will help us see how the results change over time. Additionally, the app did not collect data related to some crucial aspects of users such as age, social conditions, previous or ongoing other weight loss methods, actual physical activities that were undertaken while using the app, and information related to their diet. These factors might be significantly responsible for the observed changes. Finally, we have used self-reported measurements in the regular user group. Data in this group may be biased and pose an issue in interpreting the results.

### Conclusions

This retrospective analysis of DoFasting user data showed that users’ engagement with the app might be increased by using nudging platforms. Additionally, more engaged users tend to lose more body weight. Thus, nudging and gamified elements of mobile health apps, such as interactive tools, goals, challenges, and progress tracking, are suggested to affect engagement positively and should be investigated further in future research. Finally, the IF regime delivered through the DoFasting app might be related to increased body muscle mass and reduced body and fat mass.

## Abbreviations

AD: number of active days
CR: caloric restriction
IF: intermittent fasting
OCI: obesity class I
OCII: obesity class II
OCIII: obesity class III
TT: total time of use

## Data Availability

The data sets generated and analyzed in this study are not publicly available due to protection of confidential information of Kilo Health business and Kilo Health users, but are available from the corresponding author upon reasonable request.
